# *Clusterin*, a gene enriched in intestinal stem cells, is required for L1-mediated colon cancer metastasis

**DOI:** 10.18632/oncotarget.5360

**Published:** 2015-09-19

**Authors:** Beny Shapiro, Piera Tocci, Gal Haase, Nancy Gavert, Avri Ben-Ze'ev

**Affiliations:** ^1^ Department of Molecular Cell Biology, The Weizmann Institute of Science, Rehovot, 76100, Israel

**Keywords:** clusterin, L1, colon cancer, metastasis

## Abstract

Hyperactive Wnt signaling is a common feature in human colorectal cancer (CRC) cells. A central question is the identification and role of Wnt/β-catenin target genes in CRC and their relationship to genes enriched in colonic stem cells, since Lgr5+ intestinal stem cells were suggested to be the cell of CRC origin. Previously, we identified the neural immunoglobulin-like adhesion receptor L1 as a Wnt/β-catenin target gene localized in cells at the invasive front of CRC tissue and showed that L1 expression in CRC cells confers enhanced motility and liver metastasis. Here, we identified the clusterin (*CLU*) gene that is also enriched in Lgr5+ intestinal stem cells, as a gene induced during L1-mediated CRC metastasis. The increase in CLU levels by L1 in CRC cells resulted from transactivation of *CLU* by STAT-1. CLU overexpression in CRC cells enhanced their motility and the reduction in CLU levels in L1 overexpressing cells suppressed the ability of L1 to confer increased tumorigenesis and liver metastasis. Genes induced during L1-mediated CRC cell metastasis and enriched in intestinal stem cells might be important for both CRC progression and colonic epithelium homeostasis.

## INTRODUCTION

Aberrant activation of the canonical Wnt signaling cascade plays a key role both during early and later phases of human colorectal cancer (CRC) development [1, 2]. Wnt activation drives tumor progression by supporting the nuclear translocation of β-catenin where it forms a complex with DNA-binding proteins of the T-cell factor/lymphoid enhancer factor (TCF/LEF) family and initiates transcription of multiple target genes [3]. In recent studies, we identified β-catenin/TCF targets that support CRC invasion programs, particularly members of the neuronal immunoglobulin-like cell adhesion receptors [4, 5] of the L1 family (L1 and Nr-CAM) [6–8]. Importantly, L1 was found in populations of cells that reside at the invasive front of human CRC tissues that co-display strong nuclear β-catenin localization, indicative of Wnt pathway activation [8, 9]. Complementing these findings, the expression of L1 in human CRC cells that do not express L1 *in vitro* confers enhanced invasive activity and metastatic potential to the liver *in vivo* [10]. Our analyses of the signaling pathways that are involved in L1-mediated CRC cell metastasis indicated that the NF-κB pathway and the cytoskeletal protein ezrin are both required for conferring metastatic capacities by L1 [11]. To determine downstream targets of L1-ezrin-NF-κB signaling, we conducted a global analysis of L1-transcriptomes activated by the L1-ezrin-NF-κB pathway. We identified a number of genes that can potentially contribute to CRC progression, and in the case of one such gene, insulin-like growth factor-binding protein 2 (IGFBP-2), we showed that its expression in CRC cells mimics many of the effects conferred by L1 expression in promoting the motility and metastasis of CRC cells [12].

In the normal intestine and colon, the pit-like recessions of the epithelium, known as crypts, contain a small population of stem cells at the bottom of the crypts, and these cells are characterized by specifically expressing the *Lgr5* gene (a Wnt target gene) [13]. These cells generate all types of intestinal cell lineages in the mouse, as indicated from lineage tracing transgenic mouse studies, and an inducible activation of Wnt signaling leads to adenoma formation in Lgr5+ stem cells, strongly implicating these cells as being responsible for the initiation of CRC development [14]. Most intriguingly, we found that the L1-induced target, IGFBP-2, is localized in cells at the bottom of colonic crypts in the normal human colonic epithelium and is enriched in CRC tissues [12]. This suggested that some genes induced by L1-mediated signaling that promote CRC progression, may also play important functions in the homeostasis of cells that are localized in the stem cell compartment. To determine the significance of genes induced by L1 that are also enriched in Lgr5+ intestinal stem cells, we compared patterns of L1-induced gene expression in human CRC cells [10, 12] to recently published gene expression patterns of mouse intestinal Lgr5+ stem cells [15]. In this study we investigated one such intestinal stem cell-enriched gene, clusterin (*CLU*), whose expression is also induced by L1, and investigated its role in human CRC invasion and metastasis.

## RESULTS

### L1 induces clusterin gene expression in colon cancer cell lines independently of the NF-κB pathway

**Figure 1:**
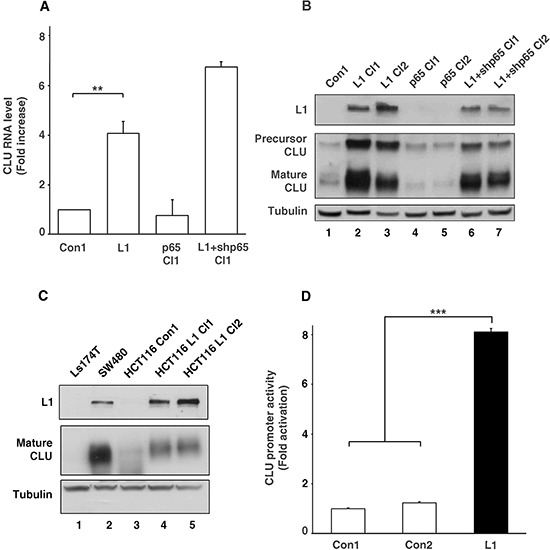
Clusterin (CLU) expression is elevated by L1 in CRC cells by *CLU* promoter activation independently of the NF-κB pathway **A.** RNA was extracted from individual cell clones isolated from stably transfected Ls174T cells with L1, control plasmid (Con1), L1 together with shRNA against p65 (L1+shp65 Cl1), and p65 alone (p65 Cl1). Quantitative RT-PCR was conducted using primers for CLU and GAPDH as control. **B.** Western blot analysis for L1, CLU and Tubulin as loading control, of the cell clones shown in A. Two cell clones of each type were used except for the control. **C.** Western blot analysis of the conditioned medium (CM) from Ls174T, SW480, HCT116 cells and HCT116 cell clones stably transfected with L1 (lanes 4 and 5). **D.** The *CLU* gene promoter reporter plasmid was co-transfected together with pSV β-galactosidase (as control vector for transfection efficiency normalization) into Ls174T CRC cells stably transfected with L1 and into two controls: a non-transfected Ls174T control and a Ls174T clone transfected with pcDNA3 (Con1 and Con2, respectively). Fold *CLU* promoter activation was determined after dividing luciferase activity by the values obtained with the empty reporter plasmid (pA3 vector). **p < 0.01, ***p < 0.001. Error bars: ±S.D.

To identify genes induced by L1 during colon cancer progression whose expression is also elevated in intestinal stem cells, we compared patterns of upregulated gene expression that we obtained from L1-overexpressing Ls174T human CRC cells [10] to those obtained from Lgr5+-mouse intestinal stem cells [15]. Such comparisons revealed a list of common genes that are induced by L1 in human CRC cells (compared to empty vector transfected cells) that are also expressed at increased levels in mouse intestinal stem cells (Table 1). We have chosen from Table 1 to study the possible involvement of clusterin (CLU) in L1-mediated CRC progression, since CLU was found to be elevated in the serum of patients in a variety of cancers including CRC [16–19] and also in tumors of the Min mouse model of activated Wnt signaling [20]. CLU is a secreted glycoprotein translated as a precursor protein of ~60kDa, followed by cleavage and glycosylation that form a secreted mature CLU of ~80kDa. The mature protein consists of two peptides linked by disulfide bonds which upon immunoblot analysis present a smear at ~40kDa, in addition to the precursor protein band at ~60kDa (Fig. 1B) [19, 21].

**Table 1:** Intestinal stem cell signature genes whose levels are upregulated in Ls174T cells stably overexpressing L1 as compared to empty vector-transfected cells

Gene symbol	Gene annotation	Gene description	Fold change	*p*-Value
HMGCS2	NM_005518	3-Hydroxy-3-methylglutaryl-coenzyme A synthase 2 (mitochondrial)	7,108	2,62E-59
CLU	NM_001831	Clusterin	2,397	2,62E-13
PLCE1	NM_016341	Phospholipase C, epsilon 1	1,874	1,57E-06
ISG20L2	NM_030980	Interferon stimulated exonuclease gene 20kDa-like 2	1,472	0,001
MCM2	NM_004526	Minichromosome maintenance complex component 2	1,441	0,001
MCM4	NM_005914	Minichromosome maintenance complex component 4	1,396	0,003
BCL11B	NM_138576	B-cell CLL/lymphoma 11B (zinc finger protein)	1,385	0,006
PPAT	NM_002703	Phosphoribosyl pyrophosphate amidotransferase	1,375	0,006
FHDC1	NM_033393	FH2 domain containing 1	1,347	0,021
PAICS	NM_001079525	Phosphoribosylaminoimidazole succinocarboxamide synthetase	1,329	0,006
CBX2	NM_005189	Chromobox homolog 2	1,303	0,025
MCM6	NM_005915	Minichromosome maintenance complex component 6	1,295	0,02
MCM7	NM_005916	Minichromosome maintenance complex component 7	1,293	0,015
RASA3	NM_007368	RAS p21 protein activator 3	1,288	0,033
CHEK2	NM_007194	Checkpoint kinase 2	1,278	0,038
MSH2	NM_000251	MutS homolog 2	1,273	0,036
DTL	NM_016448	Denticleless E3 ubiquitin protein ligase homolog (Drosophila)	1,262	0,044
MYO1B	NM_001130158	Myosin IB	1,25	0,051
CCNB1	NM_031966	Cyclin B1	1,224	0,046

To validate the results obtained from DNA gene expression microarrays, we conducted quantitative RT-PCR for CLU RNA levels and for a number of other genes shown in Table 1 (Supplemental Fig. 1) and found a significant increase in the amount of CLU RNA in L1 overexpressing CRC cells as compared to cells transfected with the empty vector (Fig. 1A). In contrast to a previous study from our laboratory indicating that many genes induced by L1-mediated signaling involve the NF-κB pathway [12], we found no increase in CLU RNA levels in CRC cells overexpressing the p65 NF-κB subunit (Fig. 1A, p65 Cl1). In addition, there was no decrease in CLU RNA levels (in fact there was an increase) in L1 overexpressing CRC cells in which the endogenous levels of p65 were suppressed using shRNA to p65 to inhibit NF-κB signaling (Fig. 1A, L1+shp65 Cl1). This increase in CLU RNA in L1 overexpressing cells was also observed when we analyzed the levels of CLU protein (both the precursor and mature forms) in Ls174T CRC cell clones overexpressing L1 (Fig. 1B, lanes 2 and 3, compare to lane 1). p65 overexpressing cells did not display an increase in CLU protein compared to control (Fig. 1B, lanes 4 and 5, compare to lane 1) and CRC cells overexpressing L1 and shRNA to p65 continued to express increased CLU levels compared to control Ls174T cells (Fig. 1B, lanes 6 and 7 compare to lane 1). The increase in CLU expression conferred by L1 in Ls174T cells was also displayed in other CRC cell lines, as seen from the analysis of CLU levels in the conditioned medium from SW480 and HCT116 CRC cells (Fig. 1C) that contains the secreted mature form of CLU protein. SW480 cells that display detectable levels of endogenous L1 also displayed high levels of CLU compared to Ls174T that do not express either L1 or CLU (Fig. 1C, lane 2 compare to lane 1). HCT116 CRC cell clones showing very low to undetectable levels of endogenous L1 (Fig. 1C, lane 3) displayed a significant increase in secreted CLU levels after their stable-transfection with L1 (Fig. 1C, lanes 4 and 5 compare to lane 3). These results demonstrate that L1-mediates the increase in CLU levels in different human CRC cell lines either expressing endogenous L1 (SW480), or after overexpression of L1 in cells with very low levels of endogenous L1 (Ls174T and HCT116). We have further shown that the increase in CLU RNA levels resulted from increased activation of the *CLU* gene promoter in L1 overexpressing Ls174T cells compared to non-transfected and empty vector-transfected Ls174T cells (Fig. 1D, Con1 and Con2 compare to L1). Together, these results suggest that L1 is inducing the transcription of CLU in a variety of human CRC cell lines, by a mechanism that is apparently independent of the NF-κB pathway.

### L1 induces *CLU* gene promoter activation via the transcription factor STAT-1

**Figure 2:**
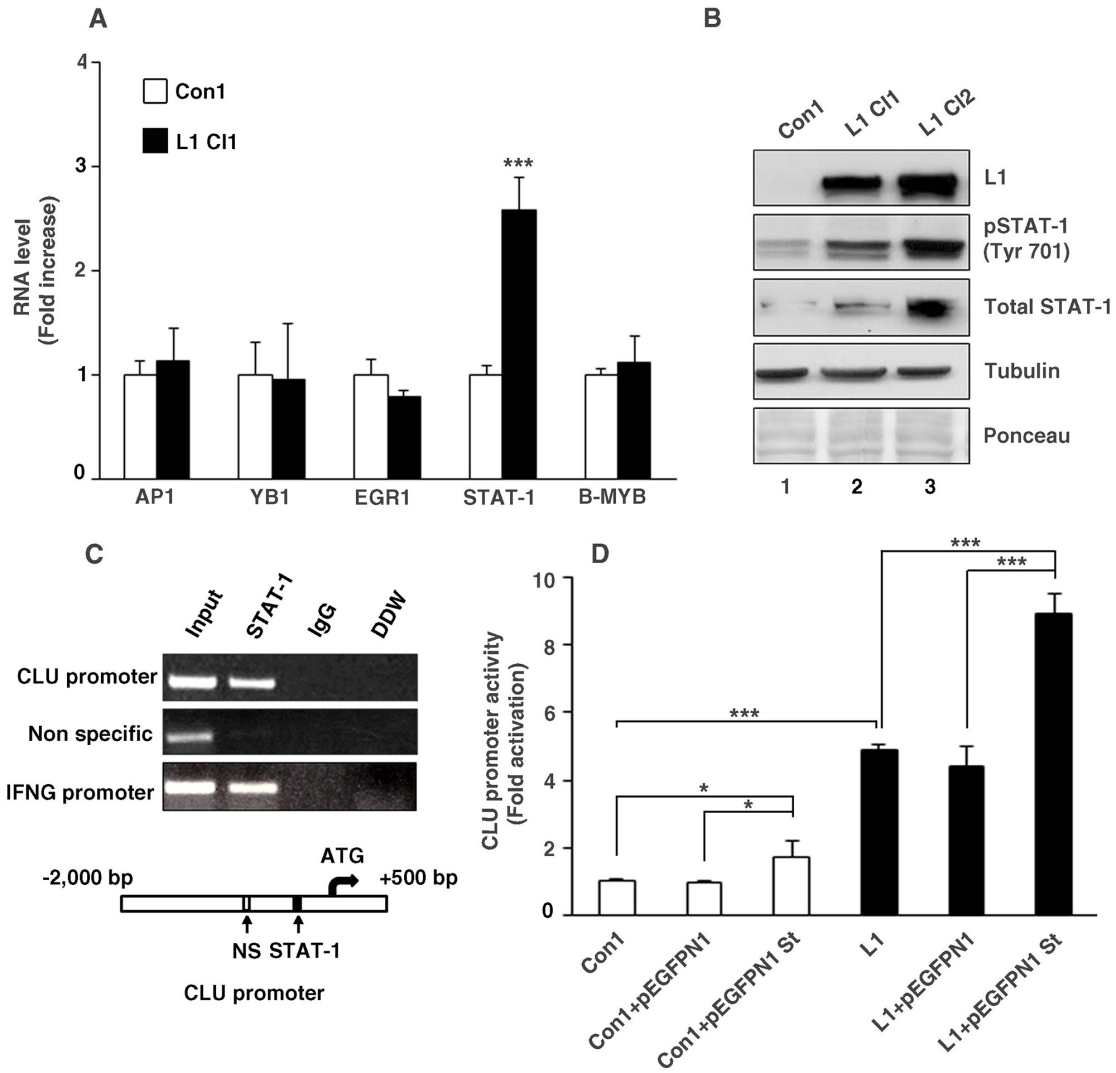
L1 activates the CLU gene promoter via an increase in STAT-1 expression **A.** Quantitative RT-PCR analysis for RNA levels of transcription factors reported to regulate *CLU* transcription (AP-1, YB1, EGR1, STAT-1 and B-MYB) in L1 overexpressing (L1 Cl1) compared to empty vector-transfected (Con1) Ls174T cells. **B.** Western blot analysis for total STAT-1 and pSTAT-1 (Tyr 701) in Ls174T CRC cell clones stably transfected with L1 (L1 Cl1 and L1 Cl2) or with the control pcDNA3 plasmid (Con1). Tubulin antibody and total protein staining of the western blots with Ponceau were used for checking equal loading. **C.** Chromatin immunoprecipitation (Ch-IP)-based PCR analysis was employed using nuclear lysates from Ls174T cells stably transfected with L1 and primer sets that amplify the STAT-1-binding sequence, a negative control (Non specific) sequence and as positive control served a sequence from the *interferon gamma* (IFNG) promoter. As a control for the PCR analysis, a PCR reaction containing double-distilled water was used (DDW). DNA fragments conjugated with nuclear proteins were immunoprecipitated with mouse anti STAT-1 or non-immune goat IgG antibodies. Fragments containing the STAT-1-binding site and the control sites were amplified from the DNA obtained by Ch-IP. **D.** The *CLU* promoter reporter plasmid was co-transfected together with the pSV β-galactosidase control vector (for transfection efficiency normalization) into an Ls174T CRC cell control clone transfected with the empty plasmid (pcDNA3) (Con1), or an L1-overexpressing CRC cell clone. These cell clones were transiently transfected with the pEGFPN1 plasmid, or the STAT-1 expression plasmid pEGFPN1 St. Fold *CLU* promoter activation was determined after dividing luciferase activity by the values obtained with the empty reporter plasmid. *p < 0.05, ***p < 0.001. Error bars: ±S.D.

To determine the transcription regulators that are involved in the L1-mediated increase in *CLU* transcription, we examined the RNA levels of various transcription factors that were reported to regulate *CLU* transactivation such as AP1 [22], YB1 [23], EGR1 [24], STAT-1 [25] and B-MYB [26] in L1 overexpressing CRC cells. The results shown in Fig. 2A summarize quantitative RT-PCR experiments determining the RNA levels of such factors in Ls174T CRC cells either overexpressing L1, or the empty vector. Among these transcription regulators, it appears that only STAT-1 RNA levels displayed a significant increase in L1 overexpressing CRC cells (Fig. 2A). This increase in STAT-1 RNA was accompanied by an increase in total STAT-1 and pSTAT-1 (Tyr 701) proteins in Ls174T CRC cell clones overexpressing L1 compared to control cells (Fig. 2B, lanes 2 and 3 compare to lane 1). Chromatin-immunoprecipitation experiments demonstrated a binding of STAT-1 to the *CLU* gene promoter and to an interferon gamma (INFG) promoter sequence that served as a positive control (Fig. 2C). Moreover, the activity of the *CLU* gene promoter was elevated in Ls174T cells by co-transfection with a STAT-1 expression plasmid, in the presence or absence of L1 (Fig. 2D). Taken together, these results suggest that L1 increases CLU levels via the transcription factor STAT-1 that binds to the *CLU* gene promoter and activates its transcription in CRC cells.

### Changes in CLU levels regulate CRC cell motility

**Figure 3:**
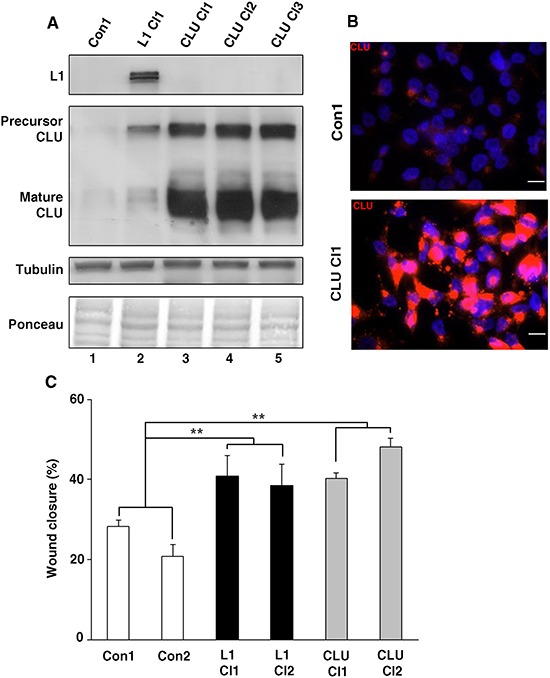
Modulation of CLU levels in CRC cells affects cell motility **A.** Ls174T CRC cell clones stably overexpressing CLU were isolated after transfection with CLU cDNA (CLU Cl1–3). The levels of CLU in selected individual clones were determined by western blotting with antibody against CLU. **B.** Immunofluorescence staining for CLU in a CLU-overexpressing CRC cell clone (CLU Cl1) and in control (pcDNA3)-transfected CRC cells (Con1) with anti-CLU antibody and with DAPI for nuclei staining. **C.** An artificial wound was introduced into a monolayer of cells, and cell motility into the wound area (after 24 h) was followed in three different wounds for each cell clone (as indicated) and expressed as percent wound closure compared to time 0 h when the wound was introduced. **p < 0.01. Error bars: ±S.D.

To study the role of increased CLU expression in human CRC progression, we isolated Ls174T CRC cell clones stably overexpressing CLU (Fig. 3A, lanes 3–5). These clones expressed high levels of both the precursor and mature forms of CLU (Fig. 3A) in the cytoplasm of the transfected cells (Fig. 3B). One of the consequences of L1 overexpression in Ls174T overexpressing cells is an increase in their motility, as measured by their ability to close an artificial wound introduced in a confluent cell monolayer (the “scratch-wound” assay) [8, 12]. The results summarized in Fig. 3C demonstrate that CLU overexpression can confer an enhanced motility that is similar in its magnitude to that induced by L1 overexpression in CRC cell clones. However, this increase in cell motility conferred by CLU overexpression in Ls174T CRC cells was not followed by a change in cell colony morphology (results not shown), E-cadherin level, or proliferation in the presence of 0.1% of serum (Supplementary Figs. 2A and 2B). Furthermore, CLU overexpressing CRC cells did not form liver metastases after injection into the tip of the spleen (Supplementary Fig. 2C), unlike Ls174T cells overexpressing L1 [10, 27] or IGFBP-2 [12].

**Figure 4:**
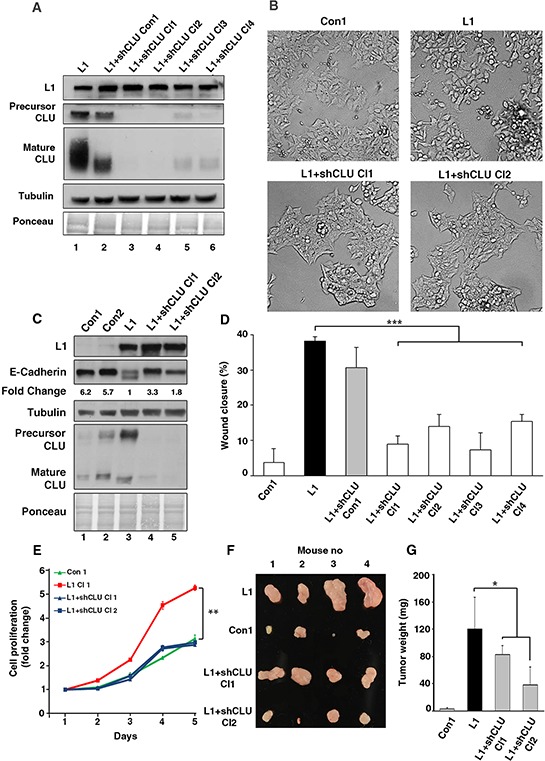
The elevated CLU expression in CRC cells overexpressing L1 is required for conferring increased motility, proliferation and tumorigenesis **A.** The level of endogenous CLU in L1-expressing Ls174T cells was suppressed by shRNA for CLU and independent cell clones displaying decreased CLU, as determined by western blot analysis with anti CLU antibody, were isolated (lanes 3–6). Lane 2 shows a clone with minimal decrease in CLU level after shCLU RNA transfection. **B.** The colony morphology of clones expressing the empty vector (Con1), L1, and two clones expressing L1+shRNA to CLU (L1+shCLU Cl1 and Cl2). **C.** The expression of E-cadherin was determined by western blot analysis in the clones described in B. **D.** The motility of the clones described in A. was determined in a “scratch-wound” experiment as described in Fig. 3C. **E.** The CRC cell clones described in B. were analyzed in triplicate cultures for their proliferation in 0.1% serum. On day 5, the proliferation of L1-expressing Ls174T cells were statistically different from all other cell clones (*p* < 0.01). **F.** The cells described in B. were also injected s.c into each mouse at four different sites in the flanks of nude mice. After 2 weeks the nude mice were sacrificed, the tumors were excised and photographed. **G.** The weight of the excised tumors was determined. *p < 0.05, **p < 0.01, ***p < 0.001. Error bars: ±S.D.

We wished to determine whether the increase in CLU expression is required for the L1-mediated increase in cell motility, and therefore isolated L1-overexpressing Ls174T CRC cell clones in which the endogenous levels of CLU were suppressed (Fig. 4A). Four cell clones overexpressing L1 were isolated in which CLU expression was dramatically suppressed (Fig. 4A, lanes 3–6), and one clone in which the level of CLU was only minimally affected by the shCLU (Fig. 4A, lane 2) and which was therefore also used as control. CRC cell clones expressing L1 in which CLU levels were suppressed displayed a more compact colony morphology (Fig. 4B, L1+shCLU Cl1 and Cl2 compare to L1) and higher levels of E-cadherin (Fig. 4C, lanes 4 and 5 compare to lane 3) as compared to cells overexpressing L1. The motile capacities of these CRC cell clones determined by the “scratch-wound” assay demonstrated a good correlation between the levels of CLU expression and cell motility in L1 expressing cells (Fig. 4D). In L1 overexpressing cells displaying significant CLU suppression, cell motility was dramatically reduced (Fig. 4D, L1+shCLU Cl1–4), while in the Ls174T cell clone overexpressing L1, but displaying only a minimal reduction in CLU level (Fig. 4A, lane 2), cell motility remained high similar to that of L1 overexpressing cells (Fig. 4D, compare L1 to L1+shCLU Con1). Together, these results imply that the increased cell motility conferred by L1 in CRC cells requires an L1-mediated elevation in the expression of CLU in these cells.

### The L1-mediated induction of CRC tumorigenesis and liver metastasis requires an increase in CLU expression

Our previous studies have shown that L1 confers an increase in the tumorigenesis and liver metastasis of CRC cells [8, 10, 27]. We wished to determine whether these properties conferred by L1 in CRC cells are dependent on endogenous CLU expression. To address these questions, we determined the proliferation of human CRC cell clones under stressful conditions (cultured in the presence of 0.1% serum) in cells overexpressing L1 and shRNA to CLU (to suppress endogenous CLU levels), and also injected immunocompromised mice s.c with these cells. Ls174T cells transfected with an empty vector and cells transfected with L1 served as negative and positive controls, respectively (see the cell clones described in Fig. 4A). The results shown in Fig. 4E demonstrate a dramatic decrease in the proliferation of L1 overexpressing CRC cells in which CLU levels were suppressed. Tumor growth in mice was determined by injection into four different locations on their flanks with the four different cell types. The tumors were excised after 2 weeks, photographed (Fig. 4F) and their weight was determined (Fig. 4G). The results summarized in Fig. 4G show that the suppression of endogenous CLU levels in L1 overexpressing cells reduced tumor growth, indicating that the increase in CLU induced by L1 was required for the more effective tumor growth that is conferred by L1 in CRC cells.

**Figure 5:**
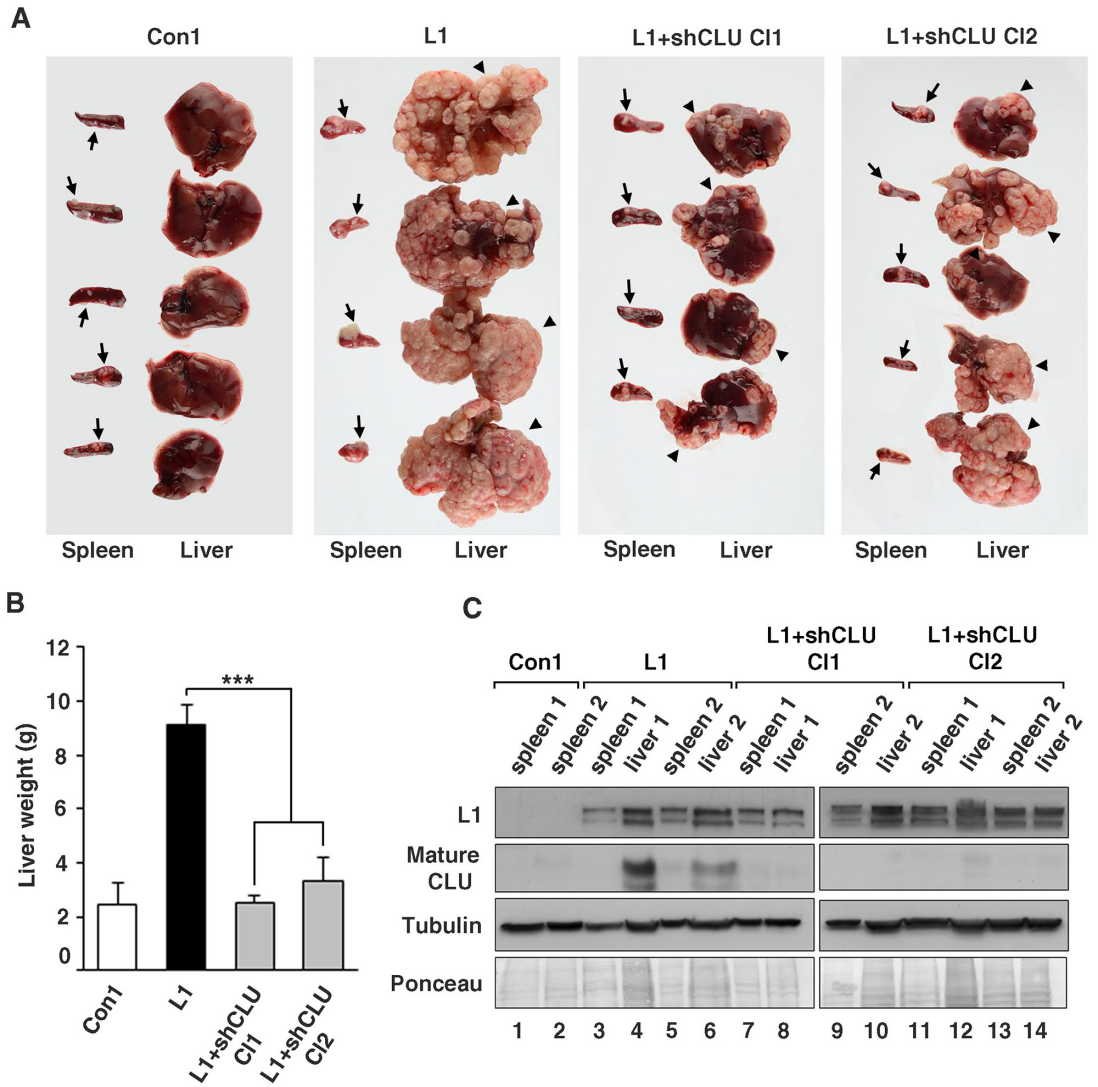
The suppression of CLU levels in CRC cells overexpressing L1 reduces their metastasis to the liver **A.** Ls174T CRC cells stably overexpressing L1, two independently isolated L1-expressing Ls174T clones in which CLU levels were suppressed by shRNA to CLU and control cells expressing the empty vector (as described in Fig. 4B), were injected into the tip of the spleen of nude mice and the formation of macrometastases in the liver was determined after 4 weeks. Arrows point to tumors formed at the site of injection in the spleen and the arrowheads point to large macrometastases in the liver. **B.** The weight of the excised livers was determined after 4 weeks. **C.** The levels of L1 and CLU proteins were determined by western blotting of tumor samples from the spleen and liver of mice to verify that the injected cell clones maintained the expression of the stably transfected L1 and the shRNA to CLU. The elevated endogenous CLU in L1 expressing cells was maintained in liver metastases (lanes 4 and 6), but there was no detectable CLU in L1+shRNA to CLU-expressing cells (lanes 8, 10, 12, 14). ***p < 0.001. Error bars: ±S.D.

While our results indicated that CLU overexpression in CRC cells is insufficient, by itself, for inducing liver metastasis (Supplementary Fig. 2C), we wished to investigate whether the ability of L1 to confer liver metastasis in CRC cells [10, 12] requires the increase in CLU expression. Using the same cells as in Figs. 4E–4G, we injected the cells into the tip of the spleen of nude mice, and the development of liver metastases was observed by excising the spleens and livers of these mice after 4 weeks (Fig. 5A). Liver metastasis was dramatic when CRC cell clones overexpressing L1 were injected into the spleen, as shown in Figs. 5A and 5B. There was a 3 to 4 fold increase in liver weight when L1 overexpressing cells were injected, compared to control cells that did not develop visible macrometastases (Figs. 5A and 5B). Suppression of CLU levels in L1 overexpressing CRC cells reduced metastasis significantly (Fig. 5A) and liver weight in such mice was similar to that of mice receiving control CRC cells (Fig. 5B). Since CLU is known for its anti apoptotic properties, we examined the percent apoptotic and necrotic cells in L1 versus L1+shCLU CRC cell clones, but did not observe significant changes in the number of apoptotic cells comparing these cell clones (Supplementary Fig. 3). These clones also did not differ in their ability to grow in soft agar (results not shown) suggesting that the effects of CLU in L1 expressing cells do not result from its anti apoptotic properties. Analysis of L1 and CLU expression in tumor tissue samples from the site of injection in the spleen and from liver metastases confirmed that these cells continued to express L1 and CLU in liver metastases in L1 overexpressing cells (Fig. 5C, lanes 4 and 6), but while continuing to express L1, they did not express CLU in mice injected with L1+shCLU Cl1 and Cl2 CRC cell clones (Fig. 5C, lanes 8, 10, 12 and 14). Taken together, these results suggest that the increased tumor growth and liver metastatic capacity conferred by L1 in CRC cells requires the elevated expression of CLU that is induced by L1.

## DISCUSSION

Cancer stem cells have been suggested to play a central role in human cancer progression [28]. In the intestine and colon, the Lgr5+ stem cells localized at the bottom of each colonic and intestinal crypt are believed to be the cells-of-origin from which intestinal and colon cancer develop [14]. Recently, a list of such Lgr5+ intestinal crypt stem-cell-enriched genes was generated from the mouse intestine [15]. In a previous study of L1-induced genes in CRC cells, we detected IGFBP-2 exclusively at the bottom of normal human colonic crypts [12], resembling the Lgr5+ stem cell compartment. We have therefore compared the gene expression pattern in such intestinal Lgr5+ stem cells from the mouse [15] to that of L1-induced genes in CRC cells [10] and generated a list of genes induced in both cell types (Table 1). From these genes, we have chosen to study the role of clusterin (CLU) in L1-mediated CRC progression. CLU is a highly glycosylated secreted protein playing a broad range of roles [29, 30] and is found in all body fluids and cell types. Being a stress-induced and pro survival protein have implicated CLU in playing a role in a variety of human tumors [19, 31–37]. In human colon cancer, the expression of CLU is considered to be a marker for tumor development and is associated with poor outcome and decreased disease-free survival [16–18, 38]. In the Min mouse model for WNT signaling-driven intestinal cancer, CLU is induced at early stages of tumor development [20]. Since an antisense oligonucleotide technology (ASO) that inhibits CLU (Custirsen, OGX-011) is currently in phase III clinical trials for castration-resistant prostate cancer patients, and in phase II clinical trials for lung and breast cancer patients [39], we wished to investigate the roles played by CLU in L1-mediated CRC cell metastasis.

We found that CLU overexpression in CRC cells is sufficient for enhancing CRC cell motility, but unlike L1 and the L1-induced *IGFBP2* gene [12], CLU overexpression, by itself, did not induce liver metastasis. Nevertheless, the ability of L1 to confer metastasis to the liver in CRC cells depends on CLU expression since a shRNA-mediated suppression of CLU in L1-overexpressing CRC cells dramatically reduced their metastatic potential. However, since L1+shCLU clones were still able to metastasize, this implies that other genes induced by L1, in addition to CLU, are required for liver metastasis. In hepatocellular carcinoma [33], lung adenocarcinoma [31] and breast cancer cell lines [32, 34, 36], CLU silencing has similarly showed a decrease in cell motility, invasion and metastasis. Studies with lung [31] and prostate cancer cells [40] suggested that an EMT-like process is involved in the CLU-mediated tumorigenic and metastatic processes. While our studies did not detect a significant change in colony morphology, or a change in E-cadherin levels upon stable overexpression of CLU in CRC cells (Supplementary Fig. 2), the loss of CLU in L1 overexpressing cells induced a more epithelial colony morphology and a mild increase in E-cadherin levels.

Since the NF-κB pathway that we have shown previously to mediate L1-signaling [11] is not involved in CLU induction (Fig. 1A and 1B), by what mechanism is L1 inducing the increase in CLU levels? Our results have shown that the increase in CLU levels in L1 overexpressing cells is induced by a STAT-1-mediated elevation in the transcriptional activity of the *CLU* promoter. Among the transcription factors reported to bind the *CLU* gene promoter, the level of STAT-1 RNA was the only one elevated after the stable-transfection of L1 into CRC cells and we detected an increase in STAT-1 protein level and phosphorylation in L1 overexpressing clones (Fig. 2B). Moreover, by Ch-IP we detected the binding of STAT-1 to the *CLU* promoter in CRC cells (Fig. 2C). This result is in agreement with the induction of a variety of cytokines and interferon-inducing genes by L1 in CRC cells [10], and suggests that such cytokines, in turn, might be responsible for the STAT-1-mediated activation of *CLU* transcription in L1-transfected CRC cells.

At present, Lgr5 expression is considered a highly reliable marker for selecting intestinal stem cells [41]. The analysis of single cells isolated from human CRC tissue also showed that a subset of cells within the tumor expressed a gene signature similar to the Lgr5+ stem cell signature [42]. A different marker, CD133 that was previously used to select cancer stem cells from human CRC tissue [43, 44], was later published to have conflicting results regarding its specificity for human CRC stem cells [45]. Supporting this notion, we found similar levels of CD133 in L1 overexpressing and control Ls174T CRC cells [9]. On the other hand, the lineage tracing studies and the exclusive localization of specific genes that are enriched in Lgr5+ stem cells at the bottom of intestinal crypts [15] provided the opportunity to study the involvement and function of such Lgr5+ cell-enriched genes in human CRC progression. Our study of the functions of CLU, which is induced in both Lgr5+ intestinal stem cells and during L1-mediated CRC metastasis, provides important information on the function of such genes during cancer progression that could also be relevant to their role in the homeostasis of the colonic epithelium. In this regard, in a recent study [47], we demonstrated that SMOC2, a gene induced in both mouse intestinal stem cells and by L1 in human CRC cells by the NF-κB-ezrin pathway, is required for L1-induced CRC cell metastasis and is localized in cells at the bottom of human colonic crypts.

In conclusion, we have shown that the L1-mediated induction of CLU, via STAT-1, has an important role in L1-mediated CRC progression. Further studies should clarify how L1 induces STAT-1 levels and the role of CLU (if any) in Lgr5+ intestinal stem cells. Using the approach presented here, it should be possible to identify additional genes with a dual role in CRC progression and intestinal stem cell biology. Such genes might prove to be useful therapeutic targets for CRC stem cells.

## MATERIALS AND METHODS

### Cell culture, motility assay and transfections

HCT116 cells were grown as described [10]. Ls174T cells were maintained in RPMI 1640 with 10% fetal calf serum. Ls174T-L1, Ls174T-p65, Ls174T-CLU, Ls174T-control cells, HCT116-L1 and HCT116-control cells were maintained in medium containing neomycin (800 μg/ml), Ls174T-L1+shp65 and Ls174T-L1+shCLU cells were maintained in medium with both neomycin (800 μg/ml) and puromycin (10 μg/ml). For motility assays, an artificial wound was introduced into confluent cell cultures using a micropipette and the percent wound closure was determined after 24 h, as described [8]. The medium was replaced with fresh medium containing 0.35 μg/ml Mitomycin-C to inhibit cell proliferation. Ls174T and HCT116 cells were transfected using Lipofectamine 2000 (Invitrogen, Carlsbad, CA, USA). For cell growth assays, 6×10^4^ cells/well were seeded in 24-well dishes and cell number determined in triplicate plates for 4 days in medium containing 0.1% serum.

### Plasmids

The wt L1 and p65 cDNAs were previously described [11]. CLU cDNA was obtained from Dr. Martin Gleave (University of British Columbia, Vancouver, British Columbia, Canada) and Dr. Saverio Bettuzzi (University of Parma, Parma, Italy). The *CLU* responsive promoter reporter plasmids were provided by Dr. Martin Gleave (see above) and Dr. David Boothman (Case Comprehensive Cancer Center, Cleveland, Ohio, USA). The p65 shRNA was previously described [27]. CLU shRNA was prepared in pSUPER.puro according to the manufacturer's instructions (pSUPER.puro RNAi System, OligoEngine, Seattle, WA, USA) using the target sequence shown in Supplementary Table 1. An additional CLU shRNA was provided by Dr. Arturo Sala (Brunel University, London, UK). The pEGFPN1 St1 expression vector was provided by Dr. Uwe Vinkemeier (School of Life Science, University of Nottingham, UK) [46].

### Determination of apoptotic and necrotic cells

Ls174T control, Ls174T-L1, Ls174T-CLU and Ls174T-L1+shCLU cells were cultured for 2 days and then incubated with FITC-labeled Annexin V (BD Biosciences, CAT-556419) and 7-aminoactinomycin D (7-AAD), 0.25 μg/1 × 10^6^ cells (BD Biosciences, CAT-559925) for 30 min followed by FACS analysis.

### Luciferase reporter assays

Ls174T-L1 and Ls174T-control cells were transiently co-transfected in triplicate plates with 0.25 μg β-galactosidase plasmid and 0.25 μg *CLU* promoter reporter plasmid in pA3, or with 0.25 μg of empty pA3 vector. HEK293T cells were transiently co-transfected in triplicate plates with 0.25 μg β-galactosidase plasmid and 0.25 μg *CLU* promoter reporter plasmid with the empty pA3 vector, or the L1 expression vector, with or without the pEGFPN1 St1 expression vector, with the empty pEGFPN1 expression vector that served as control. The medium was replenished after 24 h. Cells were lysed 48 h after transfection and luciferase and β-galactosidase levels were determined by the luciferase assay system (Promega, Fitchburg, WI, USA). Luciferase activity was normalized by β-galactosidase activity for transfection efficiency.

### Quantitative RT-PCR

RNA was isolated using the EZ-RNA kit (Biological Industries, Kibbutz Beit-Haemek, Israel). PCR was conducted using the sequences shown in Supplementary Table 1. Relative gene expression was calculated by quantitative real-time PCR (qRT-PCR) with primers designed for GAPDH, CLU, AP1, YB1, EGR1, B-MYB and STAT1 (see Supplementary Table 1). qRT-PCR was performed on the StepOnePlus™ Real-Time PCR System (Applied Biosystems, CA, USA) with the Fast SYBR^®^ Green Master Mix (Applied Biosystems, CA, USA). Primers were examined for efficiency, displaying an amplification slope of −3.33 ± 0.3 and *r*^2^ > 0.98. The PCR products were analyzed by melting curves to test their specificity. The *GAPDH* gene was used for normalization. qRT-PCR was started by incubating the samples at 95°C for 20 sec followed by PCR amplification cycles (95°C for 3 sec and 60°C for 30 sec for 40 cycles). Data analysis was conducted with the ΔΔ*C*T method with the StepOne™ software.

### Identifying intestinal stem cell signature genes upregulated by L1

Previously published DNA microarray analyses of gene expression [10] that identified genes whose RNA levels are upregulated in Ls174T colon cancer cells stably overexpressing L1 (as compared with empty vector-transfected cells), were compared to a list of 510 genes enriched in Lgr5+ mouse intestinal stem cells [15]. The list of genes upregulated under both conditions is shown in Table 1.

### Immunoblotting and immunofluorescence

Immunoblotting was carried out using the following antibodies: mouse anti-Clusterin-α (Santa Cruz Biotechnology, TX, USA, at 1:1,000), rabbit anti-Stat1α (Santa Cruz Biotechnology, TX, USA, at 1:5,000), rabbit anti-*p*-Stat1 (Santa Cruz Biotechnology, TX, USA, at 1:5,000), rabbit anti-L1 (gift from Dr. Vance Lemmon, University of Miami, FL, USA, at 1:8,000), and mouse anti-α-tubulin (Sigma-Aldrich, Rehovot, Israel, at 1:100,000). Western blots were developed by using the ECL method (Amersham Biosciences, UK). For immunofluorescence, cells cultured on glass coverslips were permeabilized with 0.5% Triton X-100 and fixed with 3% PFA. The same primary antibodies were used for immunofluorescence as for immunoblotting. The secondary antibody used was Cy3-labeled goat anti-mouse IgG (Jackson ImmunoResearch Laboratories, West Grove, PA). Images were acquired by using Eclipse E1000, Nikon objectives 60x/1.4 NA with a camera (ORCA-ER; Hamamatsu) and Volocity acquisition software (Improvision).

### Chromatin immunoprecipitation assays

Rabbit anti-Stat1α (Santa Cruz Biotechnology, TX, USA) was used for the immunoprecipitation and rabbit anti-IgG (Jackson ImmunoResearch Laboratories, Inc., West Grove, PA, USA) was used as a control antibody. Chromatin immunoprecipitation (Ch-IP) was carried out as described [12], with the exception that the DNA was purified using the PCR Purification Kit (Promega, Fitchburg, WI, USA) and subjected to PCR with the specific primers shown in Supplementary Table 1. A primers set that amplifies the STAT-1 binding sequence on the *interferon gamma* (IFNG) promoter was used as positive control. A primers set that amplifies a non-specific sequence on the *CLU* promoter was used as negative control.

### Tumor growth and metastasis assays

Tumor growth was induced by injecting 1 × 10^6^ cells s.c. in 130 μl PBS into the flanks of nude mice (5 mice per group). Control cells were injected into the opposite flank of the same mice and tumors were removed and their weight compared after 2 weeks. For metastasis assays, groups of 5 athymic (nude) mice were injected with 1.2 × 10^6^ cells in 20 μl PBS into the distal tip of the spleen. After 4–5 weeks the spleens and livers were removed as described [10].

### Statistics

Statistical significance was determined by the Fisher's exact test for mouse metastasis experiments. The significance of qRT-PCR comparisons for RNA levels was determined by ANOVA. In wound closure and luciferase reporter assay studies the significance was determined by ANOVA. A *P* value of <0.05 was considered significant and marked by an asterisk, unless otherwise indicated.

## SUPPLEMENTARY FIGURES AND TABLE


