# Supplementary material for: Clusterin, a gene enriched in intestinal stem cells, is required for L1-mediated colon cancer metastasis
Source: Oncotarget. 2015 Sep 19;6(33):34389–401. doi: 10.18632/oncotarget.5360 (PMC4741460; doi:10.18632/oncotarget.5360)
Supplement: Supplementary file 1 [file oncotarget-06-34389-s001.pdf]

SUPPLEMENTARY FIGURES AND TABLE

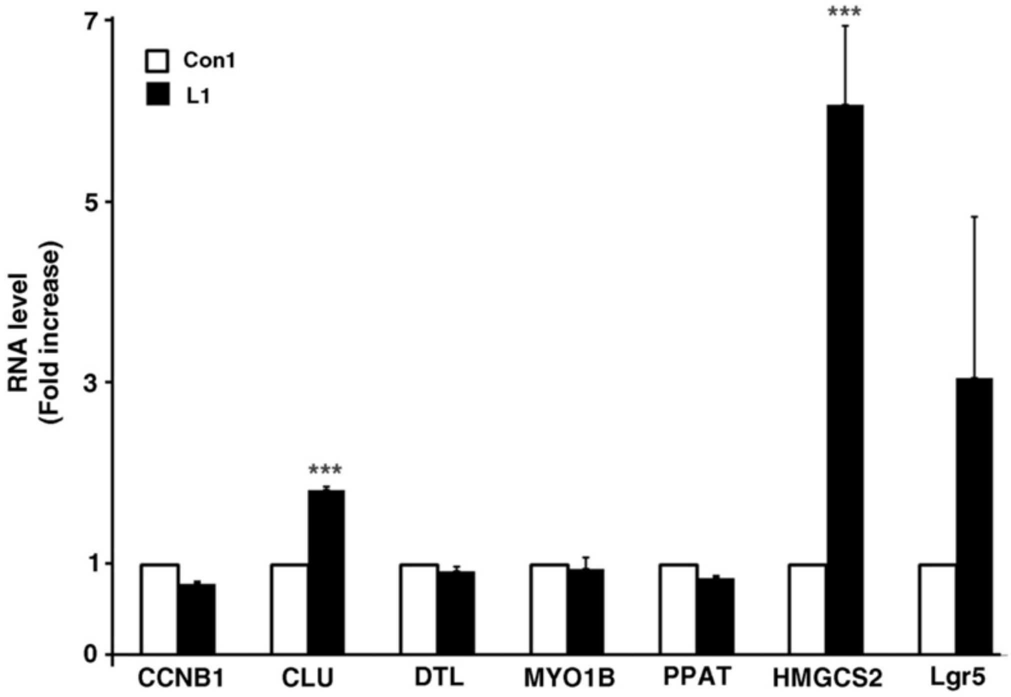

Supplementary Figure S1: Quantitative RT-PCR determination of RNA levels for some of the genes shown in Table 1. The level of Lgr5 RNA was also determined although Lgr5 did not appear in Table 1. ***$p < 0.001$. Error bars: ±S.D.

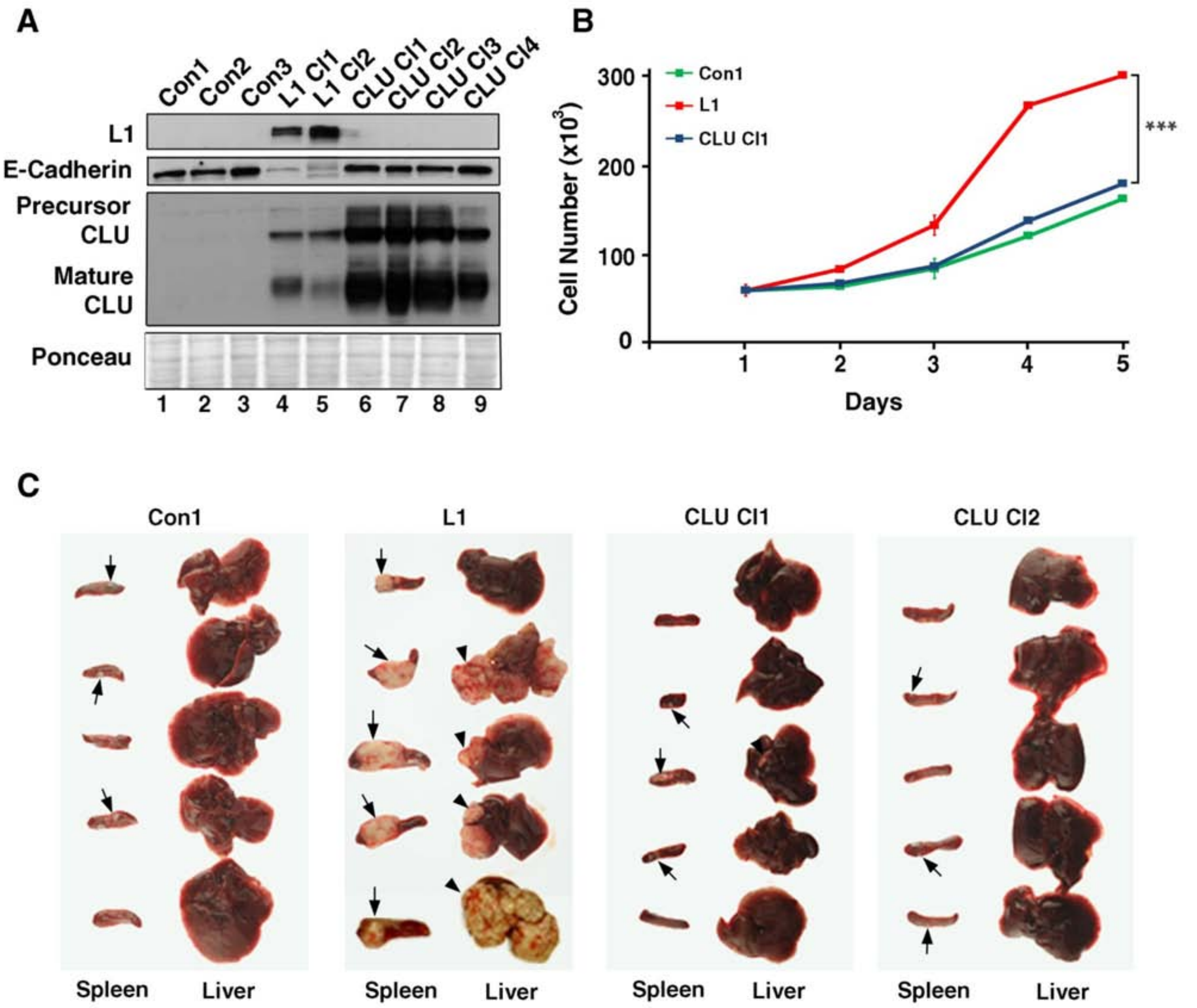

Supplementary Figure S2: Overexpression of CLU in CRC cells does not affect the proliferation of cells in low serum, E-cadherin expression, or the metastatic potential of these cells. A. Individual Ls174T CRC cell clones were isolated that either express the empty vector (pcDNA3) (Con1-3), L1 (L1 Cl1 and Cl2), or CLU (CLU Cl1-4) and the levels of L1, E-cadherin and the precursor and mature CLU were determined in these cells by western blotting with the respective antibodies. Ponceau staining of the blots was employed to examine equal loading. **B.** Cell proliferation of different cell clones as described in (A) was carried out in medium containing 0.1% serum (in triplicate plates) over 5 days and the increase in cell number was determined. **C.** The metastatic capacity to the liver of CRC cell clones described in (A) was determined by injecting cells into the tip of the spleen and following their metastasis to the liver. After 4 weeks the spleens and livers were excised and photographed. Note that only L1 overexpressing cells formed macrometastases, while cells transfected with the empty vector (Con1), or cells overexpressing CLU (CLU Cl1 and Cl2) were unable to form liver metastases. ***$p < 0.001$.

A

	Apoptotic Q2+Q4	Live Q3	Necrotic Q1
Con1	2.9%	96.3%	0.8%
L1 Cl1	2.6%	96.7%	0.7%
CLU Cl1	2.0%	97.3%	0.7%
CLU Cl2	1.1%	98.3%	0.7%
L1+shCLU Cl1	3.0%	96.2%	0.8%
L1+shCLU Cl2	2.6%	96.3%	1.1%

B

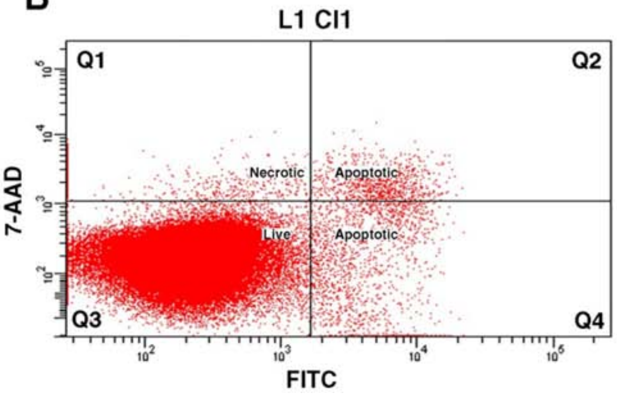

Supplementary Figure S3: Determination of the apoptotic and necrotic percentage of CRC cell clones in which the levels of L1 and CLU were modulated. A. Ls174T CRC cells stably expressing L1, CLU, or L1+shCLU, were stained with FITC-conjugated Annexin V and with 7-AAD to determine the proportion of apoptotic and necrotic cells respectively. **B.** Representative scatter scheme (of L1 overexpressing cells), showing the gating that was chosen to separate the different groups of cells (live, necrotic and apoptotic).

Supplementary Table S1: Primer sequences used in this study

Gene symbol	Forward sequence	Reverse sequence
AP1	5′-GCTGCTCTGGGAAGTGAGTT-3′	5′-TTTCTCTAAGAGCGCACGCA-3′
B-MYB	5′-CATGCTGCGTTTGTAACCCC-3′	5′-ACTTTCTTGATGGGGCTCCG-3′
CLU	5′-CAGGCCATGGACATCCACTT-3′	5′-GTCATCGTCGCCTTCTCGTA-3′
CLU (shRNA)	5′-GCGAAGACCAGTACTATCT-3′	
CLU promoter	5′-GCTATTCGTGGTGATGATGCG-3′	5′-CAAACCTGCATGACTCACGC-3′
EGR1	5′-CAGCACCTTCAACCCTCAG-3′	5′-CACAAGGTGTTGCCACTGTT-3′
GAPDH	5′-ACCACAGTCCATGCCATCAC-3′	5′-TCCACCCTGTTGCTGTA-3′
IFNG	5′-CCACTCCTCAAAAATGGCCAG-3′	5′-CATGCAAGCCAAACCTACGG-3′
Non Specific	5′-ATCCCTGGCTCCAAAATGGG-3′	5′-CAAGCAGGGTGGGCTTCAT-3′
STAT1	5′-CACGCACACAAAAGTGATGA-3′	5′-AGAGGTCGTCTCGAGGTCAA-3′
YB1	5′-GGTCATCGCAACGAAGGTTTT-3′	5′-CGCACCCTTTTCTCCTTCAAC-3′
LGR5	5’-CTGAACTAAGAACACTGA-3’	5’-TTGAGGAAGAGATGAGAT-3’
